# SUMOylation in Skeletal Development, Homeostasis, and Disease

**DOI:** 10.3390/cells11172710

**Published:** 2022-08-31

**Authors:** Huadie Liu, Sonya E. L. Craig, Vladimir Molchanov, Joseph S. Floramo, Yaguang Zhao, Tao Yang

**Affiliations:** Laboratory of Skeletal Biology, Department of Cell Biology, Van Andel Institute, 333 Bostwick Ave NE, Grand Rapids, MI 49503, USA

**Keywords:** SUMO, MSC, osteoblast, chondrocyte, osteoclast, signaling pathway, arthritis, osteosarcoma, developmental disorders

## Abstract

The modification of proteins by small ubiquitin-related modifier (SUMO) molecules, SUMOylation, is a key post-translational modification involved in a variety of biological processes, such as chromosome organization, DNA replication and repair, transcription, nuclear transport, and cell signaling transduction. In recent years, emerging evidence has shown that SUMOylation regulates the development and homeostasis of the skeletal system, with its dysregulation causing skeletal diseases, suggesting that SUMOylation pathways may serve as a promising therapeutic target. In this review, we summarize the current understanding of the molecular mechanisms by which SUMOylation pathways regulate skeletal cells in physiological and disease contexts.

## 1. Introduction

The emergence of a skeletal system was a leap forward in evolution, for it created a strong framework for the body, protecting vital organs, facilitating movement, establishing niches for hematopoiesis, and serving as a mineral reservoir. 

The skeletal system develops from mesenchymal cells originating from the ectoderm and mesoderm through one of two types of ossifications processes: intramembranous or endochondral ossification. In intramembranous ossification, mesenchymal cells directly differentiate into osteoblasts to generate the flat bones of the skull and lateral clavicles [1]. Endochondral ossification, which gives rise to the bones at the base of the skull and the long bones, starts from mesenchymal cell condensation followed by primary and secondary ossification [2]. Condensed mesenchymal cells first undergo chondrogenic differentiation to form cartilage templates [2,3,4]; next, chondrocytes in the center of the cartilage templates mature and differentiate into hypertrophic chondrocytes that secrete factors to promote vascular invasion [2,3,4,5]. This brings in hematopoietic cells from the blood and osteogenic progenitors from the perichondrium [2,3,4,5]. Next, osteoblasts, derived from either osteogenic progenitors or hypertrophic chondrocytes, produce bone matrix to replace the cartilage templates generated by the apoptotic hypertrophic chondrocytes [2,3,4,5,6,7]. At the same time, bone-absorbing osteoclasts derived from the hematopoietic lineage remodel the bone and form the bone marrow cavity [8]. Secondary ossification areas form at the center of the cartilage at both ends of long bones in a process similar to primary ossification [8,9], dividing cartilage into two parts: the growth plate, which contains growth plate chondrocytes (GPCs); and articular cartilage, which consists of articular cartilage chondrocytes (ACCs). The finely controlled, directional chondrocyte proliferation and differentiation in the growth plate propels bone elongation. The coupling between osteoblast-mediated bone formation and osteoclast-mediated bone resorption continues throughout life to maintain bone tissue homeostasis [10,11]. 

The development and homeostasis of the skeletal system require diverse and responsive signaling and cell–cell communication, which heavily rely on dynamic posttranslational modification (PTM) systems. PTMs expand the proteome size without needing de novo protein synthesis, allowing cells to regulate complex cellular processes dynamically and efficiently. PTMs participate in every aspect of cell homeostasis, and their dysregulation often leads to disease [12]. PTM pathways are common drug targets for disease treatments, for they are reversible and dependent on enzymatic activity. SUMOylation is a branch of ubiquitination-like (Ubl) PTMs that conjugate SUMO (an ~100 aa protein tag) to target proteins, with a strong connection to stress responses and aging. Below, we summarize the contribution of SUMOylation pathways to skeletal physiology and disease. 

## 2. SUMO and SUMOylation

SUMOylation is a highly dynamic and reversible PTM that attaches SUMO proteins onto target proteins. Five SUMO paralogues (SUMO1, 2, 3, 4, and 5) have been identified in mammals, each exhibiting unique expression patterns and levels of homology [13,14,15,16]. SUMO1-3 are ubiquitously expressed in all tissues, whereas SUMO4 is mainly found in kidney, spleen, and lymph nodes. SUMO5 has more restricted expression, with exceptionally high levels in testes and peripheral blood leukocytes [14,15,16,17]. In humans, SUMO2 shares 97%, 86%, 50%, and 48% amino acid sequence homology with SUMO3, 4, 5, and 1, respectively [14,15,18]. SUMO5 is 88% identical to SUMO1 [14]. 

SUMO modifications are attached to a single or multiple lysine residue(s) of target proteins (mono-SUMOylation and multi-SUMOylation, respectively). SUMO2 and 3 contain several lysine residues that are themselves SUMOylated, allowing for polymeric and branched SUMO chain formation (polySUMOylation) [14,19,20,21]. Generally, SUMO1 modifications tend to occur under normal physiological conditions, while SUMO2 and 3 conjugations are more prominent in response to stress [22], with some exceptions [23,24,25,26,27]. SUMO4 and 5 are not well characterized, and their functions remain unknown.

**Figure 1:**
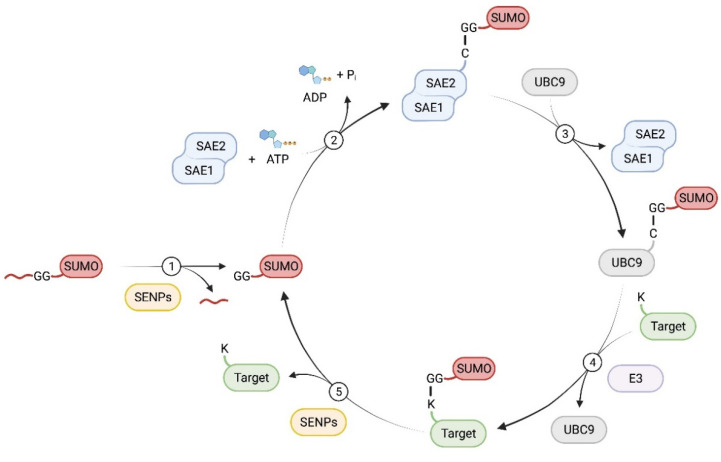
The enzymatic process of protein SUMOylation and deSUMOylation. (1) The nascent SUMO precursor protein is proteolytically cleaved to expose its C-terminal Gly-Gly motif by SENPs. (2) Mature SUMO is then activated by a heterodimer consistent of SAE1 and SAE2, the E1 complex, in an ATP dependent reaction, resulting in the formation of a thioester bond between SUMO and SAE2. (3) The activated SUMO is transferred to the E2 enzyme, UBC9. (4) With or without the help of an E3 ligase, UBC9 conjugates the SUMO group to the substrate protein by forming an isopeptide bond on a Lys residue. (5) SUMO modifications are removed from the target protein by SENPs.

SUMOylation involves a series of enzymatic reactions with E1, E2, and E3 ligases [28] (Figure 1). First, the SUMO precursor protein is cleaved by sentrin-specific proteases (SENPs), a family of SUMO-specific C-terminal hydrolases, to expose its C-terminal di-glycine (GG) motif. This mature SUMO is then activated by the E1 complex, which consists of SUMO activating enzyme subunit 1 (SAE1) and SAE2 (UBA1), by forming a thioester bond at the cysteine of SAE2 via an ATP-dependent reaction [29]. Next, the activated SUMO group is transferred to the sole SUMO E2 enzyme, UBC9 (SUMO ubiquitin-conjugating enzyme 9). Finally, UBC9, with or without the help of SUMO E3 ligases, conjugates the SUMO group to the epsilon-NH2 of a lysine in the target protein. SUMOylation substrate specificity is determined by UBC9 or SUMO E3 ligases. UBC9 recognizes consensus motifs, typically ψKxE (ψ represents a hydrophobic amino acid; K, lysine; x, any amino acid; and E, glutamic acid) [28,30]. SUMO E3 ligases facilitate the transfer of the SUMO molecule from UBC9 to the substrate proteins [28,29,31]. Unlike the ubiquitylation system, where hundreds of distinct E3 ligases have been identified, there are only a few known SUMO E3 ligases, including TRIM28, PC2, and members of the protein inhibitor of STAT (PIAS) [32,33,34,35,36]. 

In addition to proteolyzing the SUMO precursor, SENPs can also remove SUMO proteins from their targets, a process known as deSUMOylation [31]. Seven SENP proteins have been identified in humans (SENP1-3, SENP5-7, and SENP8 [19]). SENP1, 2, 3, and 5 catalyze both SUMO maturation and deconjugation, whereas SENP6 and 7 do not catalyze SUMO maturation but instead have a poly-SUMO chain-editing function [28,37,38]. Besides the SENP family, three additional SUMO proteases have been identified in humans: desumoylating isopeptidase 1 and 2 (DeSI1 and DeSI2) [39] and ubiquitin-specific protease-like 1 (USPL1) [40]. These desumoylases share little sequence homology with the SENP proteases, and their functions are less well characterized [41].

The effects of SUMO modifications on their target proteins are diverse and are mainly classified into three categories [13]: first, the attachment of the SUMO group can mask binding sites of the target protein, thus impairing its interaction with other molecules [13,42]; second, SUMOylation can introduce novel binding sites within the target protein, thus conferring novel molecular interactions [13,42]; finally, SUMO can change the structure of the target protein, thereby affecting its function [13,42]. The SUMOylation/deSUMOylation equilibrium regulates many cellular processes, including DNA damage response, mitochondrial dynamics, cell growth, proliferation, senescence, and apoptosis. Disruption of this balance is associated with many diseases, including cancer, neurodegenerative diseases, heart disease, and skeletal diseases, such as osteoarthritis (OA) and rheumatoid arthritis (RA) [29,43,44,45]. 

## 3. SUMOylation in Skeletal Cell Differentiation, Homeostasis, and Disease

Osteoblasts, chondrocytes, and osteoclasts are the major cell types of the skeletal system and cooperate seamlessly to regulate bone development and homeostasis [46,47]. 

### 3.1. SUMOylation in Osteogenesis, Osteoblast Homeostasis, and Bone Mass Regulation

**Figure 2:**
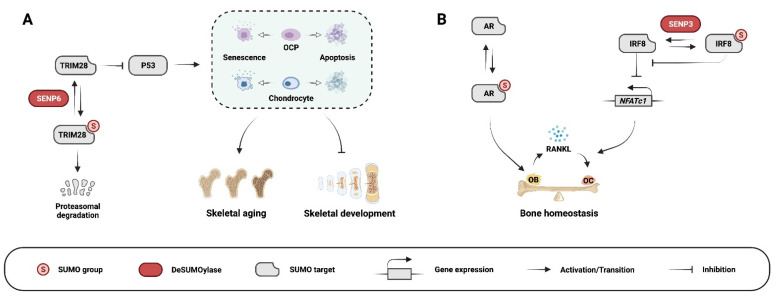
Examples of SUMOylation pathways in skeletal physiology: (**A**) SENP6 maintains proper skeletal cell homeostasis during skeletal development and aging via regulating the TRIM28/P53 axis. OCP: osteochondroprogenitor. (**B**) SUMOylation of androgen receptor (AR) increases osteoblast (OB) number and bone formation; SENP3 suppresses osteoclast (OC) formation by deSUMOylating IRF8.

SUMOylation regulates key signaling pathways and transcription factors of osteogenesis and osteoblasts; the requirement for this PTM is demonstrated by the dysregulation of bone development and homeostasis when SUMOylation is disrupted (selected examples are illustrated in Figure 2). 

We reported that postnatal and ubiquitous loss of SENP6 leads to kyphosis, a sign of premature skeletal aging [48]. Furthermore, mice with OCP-specific *Senp6* knockout have small skeletons and decreased trabecular bone mass and cortical thickness, as well as delayed secondary ossification center formation [48]. OCP-derived cell lineages lacking *Senp6* undergo severe apoptosis and cellular senescence. Mechanistically, *Senp6* loss results in excessive SUMOylation of the multifaceted protein TRIM28, which is involved in chromatin silencing, transcriptional repression, and p53 inhibition. SUMOylation destabilizes TRIM28 and weakens TRIM28-mediated p53 repression, leading to OCP/chondrocyte apoptosis and senescence [48] (Figure 2A). 

Importantly, SUMOylation regulates TGF-β/BMP signaling, a fundamental and diverse signaling network that controls embryonic skeletal development and postnatal bone homeostasis [49,50,51,52]. TGF-β/BMP superfamily ligands interact with their heteromeric receptor complexes and transmit extracellular signals to the nucleus via SMAD proteins [49,50,51,52]. In the human Saos-2 osteosarcoma cell line, SMAD4 interacts with and is SUMOylated by UBC9. Knockdown of *Ubc9* decreases the levels of SMAD4 protein and phosphorylated SMAD1, prevents the nuclear accumulation of SMAD1 and 4, and decreases the expression of osteogenic transcription factors downstream of BMP (*Runx2*, *Dlx5*, *Msx2*, and *Osx*) [53]. In contrast, *Ubc9* knockdown can elevate BMP signaling and enhance osteogenic differentiation in C2C12 mouse myoblasts and ST2 mouse bone-marrow derived stromal cells (BMSCs) [54]. Mutation of the SMAD4 SUMOylation site (K158R) increases SMAD4 transcriptional activity [54]. It is unclear why UBC9 loss has the opposite effect on BMP/SMAD signaling in these studies. One explanation would be that these cells express different SUMO E3 ligases, which SUMOylate SMAD at various sites, leading to different signaling outcomes. The loss of SUMOylation, then, would have grossly different cellular effects. This possibility needs further investigation.

Hormones and their receptors, especially the androgen receptor (AR), are important regulators of skeletal development. AR knockout dramatically reduces trabecular and cortical bone mass [55]. SUMOylation of ARs is necessary for bone mass maintenance, as mutations (K381R and K500R) within the AR SUMOylation site result in significantly decreased trabecular bone and cortical bone mass [56] (Figure 2B). Of note, while loss of AR SUMOylation decreases osteoblast numbers, the number of osteoclasts is unaffected [56].

Essential transcription factors for osteoblast differentiation, including the RUNX family members RUNX1, 2, 3, and Osterix [57,58,59,60,61,62], are also regulated by SUMOylation [63,64,65]. The SUMO E3 ligase PIAS1 promotes SUMOylation at K144 of RUNX1, K181 of RUNX2, and K148 of RUNX3 [63] and negatively regulates their functions. Increased RUNX2 SUMOylation leads to RUNX2 degradation, and PIAS1-mediated SUMOylation inhibits RUNX3 transcriptional activity [63]. Osterix SUMOylation, however, increases its activity. Osterix is SUMOylated by SUMO1 in C2C12 cells [65]. Knockdown of the SUMO E3 ligase, PIASxβ, in MC3T3-E1 mouse osteoblastic cells inhibits osteogenic differentiation and matrix mineralization [66]. PIASxβ expression enhances the transcriptional activity of Osterix, while expression of a SUMOylation-defective mutant of PIASxβ does not, suggesting that Osterix SUMOylation increases its activity [66]. 

Our own studies have shown that inhibition of SUMOylation can yield profound effects on BMSC fate determination between osteogenesis and adipogenesis. We reported that *ginkgolic acid*, a SUMOylation inhibitor that binds to E1 ligase to prevent the formation of the SAE1-SUMO intermediate, inhibits the expression of RUNX2 and Osterix while promoting the expression of the adipogenic transcription factors PPARγ and CE/BPα [67]. Consistent with our findings, PPAR-γ SUMOylation inhibits PPAR-γ transcriptional activity in BMSCs [68]. When stimulated with GDF11 (a TGFβ family member), PPAR-γ SUMOylation attenuates adipogenesis in favor of osteogenesis [68].

SUMOylation is also implicated in the epigenetic regulation of osteogenesis. In human dental follicle stem cells, SENP3 binds to and deSUMOylates RBBP5 [69], an important component of several histone methyltransferase complexes [70,71,72]. This facilitates the formation of active MLL1/MLL2 histone methyltransferase complexes that methylate H3K4 residues on the promoters of *DLX3* (an osteogenic transcription factor) and a subset of other HOX genes, thus enhancing osteogenic differentiation [69].

The above studies demonstrate that SUMOylation has crucial roles in regulating osteogenesis, osteoblast homeostasis, and bone mass via broad mechanisms, including regulation of growth factor signaling, hormone receptors, transcription factors, and epigenetic mechanisms. 

### 3.2. SUMOylation in Chondrogenesis, Chondrocyte Homeostasis, and Osteoarthritis

Chondrocytes of healthy cartilage are formed by the differentiation of skeletal progenitor/stem cells (SSCs) into GPCs through an intermediate and bipotent osteochondroprogenitor, or into ACCs via a multipotent joint progenitor [4,73,74]. GPCs proliferate and produce the extracellular matrix template for subsequent ossification, thus allowing for fast elongation of bone elements [4,73,74]. In contrast, ACCs are mostly quiescent but secrete and maintain extracellular matrix to sustain the cartilage integrity in response to outside stimuli and tissue damage and to provide a smooth and lubricated surface for articulation [75,76]. 

SUMOylation also regulates the function of chondrogenic transcription factors. SOX9, the master regulator of chondrogenesis and cartilage development [77,78,79], is a SUMO target protein. SUMOylation of SOX9 has been detected in COS-7, chick neural crest cell, U2OS osteosarcoma cells, and 293T cells; however, the consequences of SOX9 SUMOylation varies in these contexts [80,81,82,83]. A link between chondrogenesis and SOX9 SUMOylation was observed in a mouse model with OCP-specific deletion of *Shp2* [84], a protein-tyrosine phosphatase required for activating the Ras/ERK pathway [85,86]. The knock-out OCPs have increased chondrogenesis but decreased ossification [84]. Total Sox9 protein, phosphorylated SOX9, and SUMOylated SOX9 were all upregulated in SHP2-deficient chondrocytes, in addition to the SOX9 target genes, *Acan* and *Col2a1* [84]. This supports the notion that SUMOylation may regulate chondrogenesis through SOX9. 

SOX6 and NKX3.2 are two other chondrogenic transcription factors regulated by SUMOylation [87,88]. SOX6 is a downstream target of SOX9. In 293T cells, SUMOylation represses SOX6 transcriptional activity [87]. When SUMOylation is reduced, via mutations of two SOX6 SUMOylation sites, *UBC9* knockdown or loss of function mutations, or SENP2 overexpression, SOX6 transcriptional activity increases [87]. NKX3.2 regulates chondrocyte viability and differentiation, while preventing chondrocyte hypertrophy [88]. In the ATDC5 chondrogenic cell line, HDAC9-dependent deacetylation of NKX3.2 triggers its SUMOylation [88]. This leads to SUMO-targeted NKX3.2 ubiquitylation and degradation, causing hypertrophy and apoptosis of ATDC5 cells [88].

SUMOylation also likely regulates the maintenance of heterochromatin structure in articular cartilage. For instance, DGCR8 – which maintains heterochromatin through interactions with TRIM28 and HP1γ – is stabilized to prevent its degradation via the ubiquitin-proteasome pathway by SUMO1 modification at the K707 residue [89,90]. DGCR8 stabilizes heterochromatin and reduces senescence of human MSCs. Overexpression of DGCR8 alleviates OA symptoms in mice [89,90]. CLOCK, the core component of the mammalian circadian rhythm machinery, is another protein involved in maintaining heterochromatin. CLOCK transcriptional activity is increased by SUMOylation at residues K67 and K851 [91]. Overexpression of CLOCK1 prevents human MSC aging, and lentivirus-delivered CLOCK expression promotes cartilage regeneration in aged mice [92]. Future studies will need to determine whether SUMOylation of DGCR8 and CLOCK is required for regulating chondrocyte differentiation and homeostasis. Use of mouse genetic or chondrocyte models with DGCR8 or CLOCK1 SUMO sites nullified would be one way to evaluate this. 

**Figure 3:**
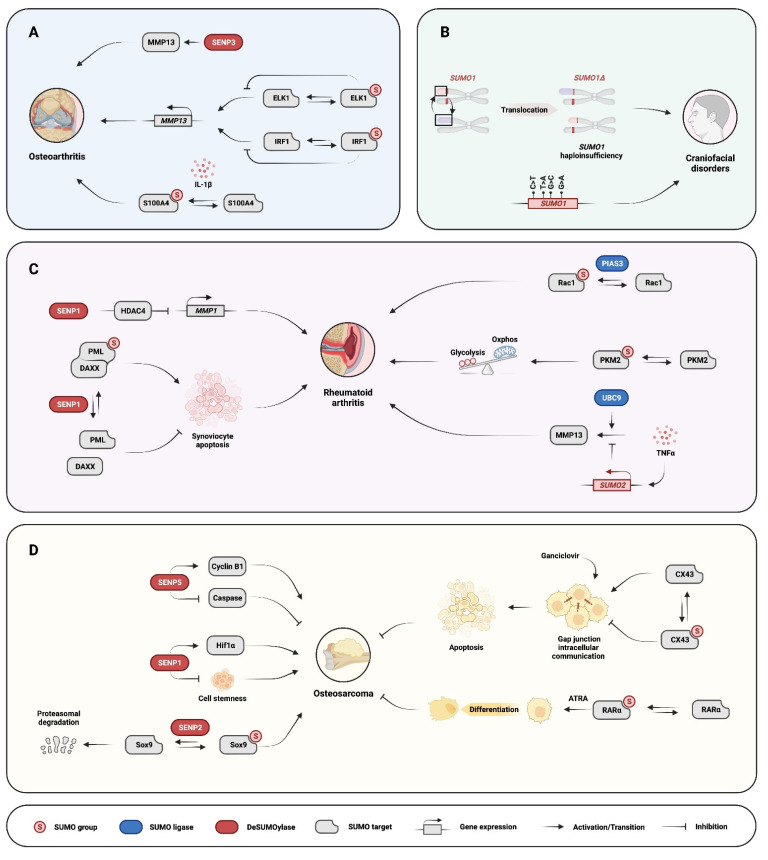
Examples of SUMOylation pathways in skeletal disease: (**A**) SENP3 overexpression upregulates osteoarthritis markers. SUMOylation of ELK1 and IRF1 prevent cartilage matrix degradation in osteoarthritis. SUMOylation of S100A4 results in the activation of *MMP13*. (**B**) A balanced chromosomal translocation that results in SUMO1 haploinsufficiency is associated with non-syndromic cleft lip with or without cleft palate (NSCLP). SUMO1 polymorphisms are associated with cleft lip with or without cleft palate, cleft palate only or NSCLP. (**C**) SENP1 prevents rheumatoid arthritis (RA) through deSUMOylating nuclear promyelocytic leukemia (PML) nuclear bodies and inhibits synoviocyte apoptosis. SENP1 attenuates rheumatoid arthritis by suppressing MMP1 expression. SUMOylation of Rac1 by PIAS3 promotes rheumatoid arthritis. SUMOylation of PKM2 induces synovial glycolysis and joint inflammation. UBC9 promotes rheumatoid arthritis by increasing the expression of *MMPs*. SUMO2 acts as an anti-inflammatory factor by preventing TNF-α stimulated expression of *MMPs*. (**D**) SENP5 is required for cell growth and apoptosis of osteosarcoma. SENP1 represses osteosarcoma by reducing the stemness of osteosarcoma cells, while promoting osteosarcoma by upregulating HIF-1α. SENP2 acts as an osteosarcoma suppressor by destabilizing SOX9. DeSUMOylation of connexin 43 (CX43) promotes gap-junction-mediated intercellular communication which sensitizes osteosarcomas cells to chemotherapy. SUMOylation of retinoic acid receptor α (RARα) is required for all-trans-retinoic acid (ATRA)-induced osteosarcoma cell differentiation.

Osteoarthritis (OA) is a disease that is characterized by progressive loss of cartilage, the formation of bone spurs, and chronic synovial inflammation [93]. OA severely impairs joint function and often causes joint pain [93]. The onset and progression of OA are associated with various risk factors, including gender, genetic predisposition, obesity, joint malalignment, sports injury, and aging [93]. Several lines of evidence suggest that enhanced SUMOylation promotes OA pathogenesis. A large genome-wide association analysis in Europe identified the rs9350591 C/T single nucleotide polymorphism (SNP), located upstream of the *SENP6* locus, as one of the most strongly OA-associated SNPs [94]. SENP6 expression is significantly decreased in OA cartilage even in the absence of rs9350591, suggesting that a deficiency in SENP6 desumoylase activity may be a widespread phenomenon in OA [95]. Further support for SUMOylation promoting OA comes from studies where IL-1β treatment of human ACs was shown to induce SUMO1 modification of S100A4 (a member of the Ca^2+^-binding S100 proteins that modulates p53 transcriptional activity), resulting in S100A4 nuclear translocation. Nuclear S100A4 binds to the *MMP13* promoter region and increases expression of a major OA-associated protease that degrades cartilage, MMP13 [96] (Figure 3A). 

In contrast, several studies have found that SUMOylation decreases OA marker expression. A high-throughput screen of primary human ACCs identified *SENP3* as a pro-OA gene [97]. SENP3 overexpression up-regulated several OA markers, including *MMP13*, *COX2* (cyclooxygenase-2), *iNOS* (inducible nitric oxide synthase), and *AGG1* (aggrecanase-1) [98] (Figure 3A). Additionally, SUMO1 modification of interferon regulatory factor 1 (IRF-1) was induced by the antioxidant alpha-lipoic acid in human ACCs [98]. This modification decreased the transcriptional activity of IRF-1, thus reducing the IL-1β-induced expression of OA marker genes, including *MMP3* and *MMP13* [99] (Figure 3A). Furthermore, in human primary ACCs, basic fibroblast growth factor (bFGF) increases ETS-like-1 protein (ELK-1) phosphorylation but decreases ELK-1 SUMOylation. Decreased ELK-1 SUMOylation enhances its transcription of MMP13, thus promoting cartilage matrix degradation [100] (Figure 3A). 

### 3.3. SUMOylation in Osteoclastogenesis and Osteoclast Function

Osteoclasts differentiate from the hematopoietic cell lineage upon induction by cytokines, such as m-CSF and RANKL, present in the bone and bone marrow microenvironment [101,102]. Osteoclast progenitors differentiate, fuse, and form multinucleated mature osteoclasts, which produce acid and matrix-degrading proteases and serve as dedicated bone-resorbing cells of the skeletal system [101,102]. 

Recent studies revealed the regulatory role of SUMOylation in osteoclast formation and function. For instance, SENP3 suppresses osteoclastogenesis. Mice with the *Lyz2*-Cre-mediated *Senp3* deletion in bone marrow-derived monocytes exhibit decreased bone mass [103]. These knockout mice also have aggravated bone loss after ovariectomy due to overactivation of osteoclasts. Mechanistically, *Senp3* deletion increases SUMO3 modification of IRF8 and weakens the ability of IRF8 to suppress *NFATc1* (a master regulator of osteoclastogensis) expression [103] (Figure 2B). A similar effect on osteoclastogenesis is observed in transgenic mice overexpressing the SUMO E3 ligase PIAS3 [104]. The mice exhibit an osteopetrotic phenotype caused by impaired osteoclast differentiation [104]. PIAS3 overexpression inhibited *c-Fos* and *Nfatc1* expression in RAW264.7 cells, thereby blunting RANKL-induced osteoclastogenesis [104]. Likewise, in a bone marrow monocyte–osteoblast co-culture system, PIAS3 overexpression in osteoblasts downregulated IL6-induced RANKL expression and inhibited osteoclast formation. Downregulation of PIAS3 in osteoblasts in the same system increased RANKL expression [104]. Thus, PIAS3 inhibits osteoclastogenesis either by intrinsically inhibiting osteoclast differentiation or by indirectly suppressing the expression of osteoclastogenic cytokines, such as RANKL, from osteoblasts. However, as PIAS3 can also affect transcriptional regulators (such as NFκB and STAT3 signaling [105]) independent of SUMO ligase activity, it is unclear whether the activity of PIAS3 in osteoclastogenesis depends upon its E3 ligase function or not. 

### 3.4. SUMOylation in Developmental Diseases

#### 3.4.1. Split Hand/Split Foot Malformation (SHFM)

SHFM is a rare limb malformation characterized by clefts in the middle of the hands and feet, as well as syndactyly and aplasia/hypoplasia of phalanges, metacarpals, and metatarsals [106]. P63α mutations are associated with SHFM [107,108]. Notably, SUMO1 is conjugated to K549 and K637 of P63α, following the binding of UBC9 to the C-terminal domain of P63α [109,110]. The SHFM-associated P63α mutation, Q634X, disrupts the interaction between P63α and UBC9. K549E and K637E mutations of P63α, both of which block P63α SUMOylation, markedly increase the transcriptional activity of TAP63α (an isoform of P63α containing the N-terminal transactivation domain) [110]. At the same time, these mutations inhibit the dominant-negative effect of the naturally occurring N-terminus truncated isoform of P63α, ΔNP63α. Both SUMOylation and ubiquitylation are required for the efficient degradation of ΔNP63α [111]. One downstream molecular consequence of loss of P63α SUMOylation was determined using cells expressing mutant P63α lacking the two SUMOylation sites. In these cells, the expression of genes related to bone and tooth development, such as *Runx2* and *Mint* [110], were decreased. These data emphasize the functional importance of SUMOylation of P63α in limb development.

#### 3.4.2. Craniofacial Disorders

Craniofacial disorders are one of the most common human birth defects. Cleft lip and palate are the most frequent types of craniofacial disorders [112], and several studies have linked SUMO1 deficiency to these disorders. First, a balanced chromosomal translocation 46,XX,t(2;8)(q33.1;q24.3) resulting in *SUMO1* haploinsufficiency was identified in a patient with isolated cleft lip and palate [113] (Figure 3B). Second, a 4-SNP SUMO1 haplotype was found significantly associated with non-syndromic cleft lip with or without cleft palate (NSCLP) from a study of 181 patients and 162 healthy controls of Han Chinese origin [114]. Other studies have also related SUMO1 to cleft lip with or without cleft palate, cleft palate only, or NSCLP in Poland [115], Ireland [116], and western China [117] (Figure 3B). In addition, transcription factors such as TBX22, MSX1, SATB2, P63, PAX9, TRPS1, and EYA1, which contribute to the development of the lip and palate, have all been identified as substrates of SUMO modification [118]. For example, SUMOylation regulates the subnuclear localization, stability, and transcriptional activity of SATB2, affects subnuclear localization of MSX1, modulates the transcriptional activity and stability of P63 (see above section on SHFM), facilitates the transcriptional repressor activity of TBX22, and regulates the transcriptional suppression function of TRPS1 [119,120,121,122,123,124]. In summary, the formation of the lip and palate appears to be particularly sensitive to changes in SUMOylation [118].

### 3.5. SUMOylation in rheumatoid arthritis

Rheumatoid arthritis (RA) is a chronic systemic, inflammatory disease characterized by joint stiffness and destruction [125,126]. Synovial inflammation is a hallmark of RA and the main driver of cartilage degradation. The main cellular features of RA include synovial hyperplasia, increased vascularity, and inflammatory cell infiltration [125,126]. 

A direct relationship between the SUMOylation pathway and RA was first reported in 2000 [127]. SUMO1 mRNA was found to be highly expressed in synovial specimens from RA patients, predominantly in the synovial fibroblasts of the lining layer and at the sites where cartilage is invaded by synovium [127]. The expression of SUMO1 in RA synovial fibroblasts (RASFs) is over 30 times higher than that found in OA synovial fibroblasts or normal fibroblasts [127]. Furthermore, the expression of the E3 ligase PIAS3 is also increased in RAFLSs and RA synovial tissues [128]. A recent study found that SUMO1 knockdown inhibits the migration and invasion of RA fibroblast-like synoviocytes (RAFLSs) and RAFLS expression of *MMP1* and *MMP3.* Mechanistically, SUMO1 deficiency suppresses the activity of the Rac1/PAK1 pathway, which normally promotes cell motility [129]. PIAS3 promotes the SUMOylation of Rac1 and activates the expression of Rac1 downstream targets, such as PAK1 and JNK [128]. Decreased PIAS3 expression in RAFLS also inhibits the invasion and migration of RAFLSs and the expression of *MMP3*, *MMP9*, and *MMP13* [128] (Figure 3C). 

SUMO E1 conjugating enzymes SAE1 and SAE2 are also increased in FLSs and synovial tissues of RA patients [130]. Knockdown of SAE1 or SAE2 by siRNA results in a less aggressive phenotype and reduced inflammation of RAFLSs [130]. SAE1- and SAE2-mediated SUMOylation of pyruvate kinase M2 (PKM2) promotes its phosphorylation and nuclear translocation, resulting in the suppression of pyruvate kinase activity, contributing to synovial glycolysis and joint inflammation [130](Figure 3C).

Further support for increased SUMOylation in RA comes from the finding that expression of the SENP1 desumoylase is decreased in RASFs [131,132]. Mechanistic studies revealed that overexpression of SENP1 can deSUMOylate nuclear promyelocytic leukemia (PML) nuclear bodies and inhibit the recruitment of DAXX, a FADD (Fas-associated death domain)-interacting protein, to PML nuclear bodies, thus promoting the Fas-mediated apoptosis of RASFs [131] (Figure 3C). In addition, SENP1 suppresses *MMP1* expression by promoting HDAC4 binding to the *MMP1* promoter, further weakening the invasiveness of RASFs [132] (Figure 3C). 

These studies show that increased SUMOylation is positively related to RA, suggesting that downregulation of SUMOylation may have therapeutic benefits. In support of this, in a mouse collagen-induced arthritis model, downregulation of UBC9 using siRNA can reduce arthritis intensity scores and joint destruction [133]. RA-related markers, including serum levels of anti-collagen (CII) antibodies, VEGF-A, MMP3, and MMP9, were also decreased. Moreover, downregulating UBC9 expression in ex vivo human RAFLS cultures inhibits TNF-α-stimulated secretion of VEGF-A, MMP-3, and MMP-9, and blocks RAFLS proliferation and migration [133] (Figure 3C). 

The only study supporting the anti-RA function of SUMOylation reported that the expression of SUMO2 in RA tissue or RASFs is significantly higher than that of OA tissues and is increased in the synovium and synovial fibroblasts of human TNF-transgenic (hTNFtg) mice, a common RA model [134]. TNF-α treatment promotes the expression of SUMO2 in vitro, while *SUMO2* knockdown significantly increases the expression of *MMP3* and *MMP13* induced by the TNF-α- and IL-1β-stimulated NF-κB pathway [134] (Figure 3C). 

Most of these studies show that gross alteration of SUMOylation in the joint contributes to the development of OA and RA. Although the detailed mechanisms are still not well understood, some insight may be derived from studies in other disease conditions or cell types, which suggest that SUMOylation regulates inflammation by modulating the NFκB pathway, the PPARγ pathway, among others [68,135,136,137,138].

### 3.6. SUMOylation in Osteosarcoma

Osteosarcoma is the most common cancer type in the human skeletal system. It occurs in humans in a biphasic pattern, i.e., with a peak in adolescence and another in patients over 60 years of age [139,140]. SUMOylation of proteins has a crucial role in regulating the cell cycle, genome stability, and the expression of oncoproteins and tumor suppressors [141,142], and has been linked to the development of osteosarcoma [82,143,144,145,146,147,148,149,150,151] (Figure 3D). However, there is no consensus on whether SUMOylation is pro- or anti-tumorigenic in osteosarcoma, as this is likely dependent on the specific proteins and/or the specific SUMOylation site within them that are SUMOylated.

#### 3.6.1. Studies Supporting a Pro-Tumorigenic Effect of SUMOylation

Several studies have linked increased SUMOylation to osteosarcoma. For example, *UBC9* is overexpressed in osteosarcoma tissues and cell lines [143]. *UBC9* knockdown inhibits the proliferation and migration of osteosarcoma cells and markedly increases the sensitivity of these cells to the combination treatment of herpes simplex virus thymidine kinase/ganciclovir (HSV-TK/GCV) [143]. The integrity of gap-junction-mediated intercellular communication (GJIC) is required for the HSV-TK/GCV-induced tumor repression. Ubc9 knockout decreases SUMO1 modification and increases the free protein level of connexin 43 (CX43), a component of gap junctions [143]. Thus, UBC9 deficiency sensitizes osteosarcoma cells to chemotherapy by reconstructing and promoting GJIC [143] (Figure 3D). 

In addition, SENP1 expression is decreased in osteosarcoma tissues, cell lines, and osteosarcoma stem cells compared to non-cancer cells and stem cells [144]. Low SENP1 is essential for maintaining the stemness of osteosarcoma stem cells, and overexpression of SENP1 markedly decreases the stemness of osteosarcoma cells while sensitizing them to apoptosis induced by HSV-TK/GCV combination treatment [144] (Figure 3D). This highlights the potential for using SENP1 activation for the treatment of osteosarcoma. SENP2 expression is also significantly decreased in osteosarcoma compared with adjacent normal tissue [82]. SENP2 overexpression inhibits osteosarcoma cell proliferation, migration, and invasion, while SENP2 knockout by CRISPR-Cas9 has the opposite effect [82]. Mechanistically, SENP2-mediated deSUMOylation promotes SOX9 ubiquitylation and degradation [82]. SOX9 knockdown greatly reduces the proliferation and invasiveness of the SENP2 knockout osteosarcoma cells [82]. This study suggests that SENP2 acts as an osteosarcoma suppressor by destabilizing SOX9 (Figure 3D). 

Talin is a key component of focal adhesions [152] and can be modified by SUMOylation in U2OS osteosarcoma and MDA-MB-231 breast cancer cells. Using ginkgolic acid (GA) to inhibit SUMOylation increases the number and size of talin-containing focal adhesions [145]. Inhibition of SUMOylation can significantly reduce the migration of MDA-MB-231 breast cancer cells, but this effect was not studied in U2OS cells [145]. Cumulatively, these studies indicate that SUMOylation can promote osteosarcoma proliferation, invasion, and migration, and that targeting it may be a relevant point of therapeutic intervention.

#### 3.6.2. Studies Supporting an Anti-Tumorigenic Effect of SUMOylation

In contrast to what was presented above, several studies suggest that SUMOylation can have *anti-osteosarcoma* effect. For example, the desumoylase SENP5 is highly expressed in osteosarcoma cells and tissues [146]. Silencing SENP5 expression in two osteosarcoma cell lines, U2OS and Saos-2, significantly inhibits growth and colony formation and promotes apoptosis [146]. The inhibition of osteosarcoma growth following *SENP5*-knockdown is likely via an increase in caspase-3/-7 activity (apoptosis activators) and a decrease in the expression of the cell cycle gene, cyclin B1 [146] (Figure 3D). 

The expression of the E3 ligase, PIASxα, is lower in osteosarcoma compared to adjacent tissue [147]. Notably, PIASxα overexpression can significantly inhibit osteosarcoma cell proliferation and increase apoptosis [147], whereas PIASxα silencing in U2OS cells increases the expression of cyclin D kinase genes. Moreover, PIASxα overexpression weakens the tumorigenic potential of U2OS cells in nude mice [147]. Because of the aforementioned pleiotropic functions of PIASxα, further studies are needed to determine whether the anti-tumor effects observed here depend on SUMO-E3 ligase activity of PIASxα. 

As another example, all-trans-retinoic acid (ATRA), is an anti-cancer drug that induces osteosarcoma cell differentiation, which is used as a prognostic indicator of weakened osteosarcoma malignancy and tumor progression [148]. SUMO1 deletion blocks the anti-osteosarcoma efficiency of ATRA, demonstrating that SUMO1 is required for the pro-differentiation effect of ATRA [149]. In addition, the target of ATRA, retinoic acid receptor α (RARα), is stabilized by SUMOylation at K399 [149]; and mutation of K399 of RARα impairs ATRA-induced osteosarcoma cell differentiation [149]. These suggest that SUMO1 acts as an anti-osteosarcoma molecule by targeting RARα (Figure 3D). 

In a recent study, higher expression of SENP1 was found in human osteosarcoma tissue than in adjacent normal tissue (53/60 vs. 28/60) [151]; and levels of SENP1 derived from patient plasma exosomes directly correlate with osteosarcoma tumor size, location, necrosis rate, pulmonary metastasis, and surgical stage [151]. Furthermore, patients with higher plasma levels of exosome-derived SENP1 had worse tumor malignancy and overall survival rate. Notably, the prognostic value of plasma exosome-derived SENP1 levels in osteosarcoma was found to be better than plasma SENP1 [151]. In the human osteosarcoma cell line MG-63, a hypoxic environment (similar to that typically found in tumors) induces the expression of high amounts of SENP1 [150], and inhibiting SENP1, in turn, reduces the expression of two major hypoxia-induced genes, *HIF1α* and *VEGF* (vascular endothelial growth factor). This reduction in HIF1α normalizes hypoxia-induced SENP1 expression [150]. SENP1 knockdown accelerates apoptosis by decreasing *Bcl-2* expression while increasing *Bax* expression. The ultimate effect of this is the reduction of MG-63 cell invasiveness by suppressing epithelial-mesenchymal transition (EMT) genes [150]. These findings suggest a positive feedback loop between SENP1 and HIF1α in regulating proliferation, invasion, and EMT of osteosarcoma cells in hypoxic conditions (Figure 3D). 

In summary, changes in both SUMOylation and deSUMOylation enzymes are related to osteosarcoma. UBC9, the only SUMO E2 enzyme, promotes osteosarcoma, suggesting that a global increase in SUMOylation favors osteosarcoma development. However, several SENP deSUMOylases show diverse roles in osteosarcoma, reflecting that the SUMOylation status of their specific targets are the determinants of osteosarcoma development. While we could not make a simple generalization whether SUMOylation is osteosarcoma-promoting or -inhibiting, these studies demonstrate SUMOylation as a crucial PTM in osteosarcoma tumor initiation and progression. 

### 3.7. SUMOylation in Chondrosarcoma

SUMOylation is also associated with malignant tumors that form from bone cartilage, known as conventional chondrosarcoma [139,153]. SUMO1 and SUMO2/3 expression are positively correlated with increased aggressiveness of chondrosarcomas, and patients with high SUMO2/3 expression have poorer survival outcomes [153]. The authors of that study suggest that SUMO expression could be a useful prognostic marker in chondrosarcoma. Future studies are warranted to explore this and to identify the SUMOylation targets that promote chondrosarcoma development. 

## 4. Summary and Future Perspectives

PTM by SUMOylation regulates signaling pathways and transcription factors that are crucial for skeletal cell differentiation, development, and homeostasis (Figure 2). Dysregulation of SUMOylation is associated with skeletal diseases, such as OA and RA, craniofacial defects, and bone tumors (Figure 3). Thus, targeting SUMOylation/deSUMOylation pathways is a promising strategy for the development of new treatments for these disorders. However, this requires a better characterization of the SUMOylation/deSUMOylation machinery and identifying regulators and effectors (substrates) of SUMOylation/deSUMOylation. The establishment of tissue- and disease-specific mouse genetic models that manipulate the expression of SUMO pathway components in skeletal cells will be a valuable resource to achieve this goal.

Targeting SUMOylation for disease treatments is in a more preliminary state than that of targeting ubiquitination, but some basic studies suggest that this is a promising pursuit. For example, the SUMO E1 inhibitor ML792 reduces proliferation, viability, and colony formation of chondrosarcoma cell lines [153]. The SUMOylation inhibitor of TRPS1 (a transcriptional repressor of genes important for bone and cartilage development and maintenance), GSK145A(23), has been developed to treat bone and cartilage diseases [154]. A novel SENP1 inhibitor, senpPNA-R8, is also being investigated in a clinical trial for treating osteosarcoma [155]. 

Finally, SUMOylation is involved in regulating stress response, epigenetics, and senescence, all of which are closely associated with aging. Future studies dissecting the relationship between SUMOylation and aging and tissue rejuvenation will likely also bring forth new approaches to promote skeletal health.

## References

[1] Percival C.J., Richtsmeier J.T. (2013). Angiogenesis and intramembranous osteogenesis. Dev. Dyn..

[2] Long F., Ornitz D.M. (2013). Development of the endochondral skeleton. Cold Spring Harb. Perspect. Biol..

[3] Ono N., Ono W., Nagasawa T., Kronenberg H.M. (2014). A subset of chondrogenic cells provides early mesenchymal progenitors in growing bones. Nat. Cell Biol..

[4] Kronenberg H.M. (2003). Developmental regulation of the growth plate. Nature.

[5] Wan C., Shao J., Gilbert S.R., Riddle R.C., Long F., Johnson R.S., Schipani E., Clemens T.L. (2010). Role of HIF-1alpha in skeletal development. Ann. N. Y. Acad. Sci..

[6] Zhou X., von der Mark K., Henry S., Norton W., Adams H., de Crombrugghe B. (2014). Chondrocytes transdifferentiate into osteoblasts in endochondral bone during development, postnatal growth and fracture healing in mice. PLoS Genet..

[7] Yang L., Tsang K.Y., Tang H.C., Chan D., Cheah K.S. (2014). Hypertrophic chondrocytes can become osteoblasts and osteocytes in endochondral bone formation. Proc. Natl. Acad. Sci. USA.

[8] Mackie E.J., Ahmed Y.A., Tatarczuch L., Chen K.S., Mirams M. (2008). Endochondral ossification: How cartilage is converted into bone in the developing skeleton. Int. J. Biochem. Cell Biol..

[9] Morini S., Continenza M.A., Ricciardi G., Gaudio E., Pannarale L. (2004). Development of the microcirculation of the secondary ossification center in rat humeral head. Anat. Rec. A Discov. Mol. Cell Evol. Biol..

[10] Novack D.V., Teitelbaum S.L. (2008). The osteoclast: Friend or foe?. Annu. Rev. Pathol..

[11] Raggatt L.J., Partridge N.C. (2010). Cellular and molecular mechanisms of bone remodeling. J. Biol. Chem..

[12] Liu Y., Molchanov V., Yang T. (2021). Enzymatic Machinery of Ubiquitin and Ubiquitin-Like Modification Systems in Chondrocyte Homeostasis and Osteoarthritis. Curr. Rheumatol. Rep..

[13] Wilkinson K.A., Henley J.M. (2010). Mechanisms, regulation and consequences of protein SUMOylation. Biochem. J..

[14] Liang Y.C., Lee C.C., Yao Y.L., Lai C.C., Schmitz M.L., Yang W.M. (2016). SUMO5, a Novel Poly-SUMO Isoform, Regulates PML Nuclear Bodies. Sci. Rep..

[15] Bohren K.M., Nadkarni V., Song J.H., Gabbay K.H., Owerbach D. (2004). A M55V polymorphism in a novel SUMO gene (SUMO-4) differentially activates heat shock transcription factors and is associated with susceptibility to type I diabetes mellitus. J. Biol. Chem..

[16] Guo D., Li M., Zhang Y., Yang P., Eckenrode S., Hopkins D., Zheng W., Purohit S., Podolsky R.H., Muir A. (2004). A functional variant of SUMO4, a new I kappa B alpha modifier, is associated with type 1 diabetes. Nat. Genet..

[17] Han Z.J., Feng Y.H., Gu B.H., Li Y.M., Chen H. (2018). The post-translational modification, SUMOylation, and cancer (Review). Int. J. Oncol..

[18] Saitoh H., Hinchey J. (2000). Functional heterogeneity of small ubiquitin-related protein modifiers SUMO-1 versus SUMO-2/3. J. Biol. Chem..

[19] Kunz K., Piller T., Muller S. (2018). SUMO-specific proteases and isopeptidases of the SENP family at a glance. J. Cell Sci..

[20] Matic I., van Hagen M., Schimmel J., Macek B., Ogg S.C., Tatham M.H., Hay R.T., Lamond A.I., Mann M., Vertegaal A.C.O. (2008). In vivo identification of human small ubiquitin-like modifier polymerization sites by high accuracy mass spectrometry and an in vitro to in vivo strategy. Mol. Cell Proteom..

[21] Hendriks I.A., D’Souza R.C., Yang B., Verlaan-de Vries M., Mann M., Vertegaal A.C. (2014). Uncovering global SUMOylation signaling networks in a site-specific manner. Nat. Struct. Mol. Biol..

[22] Pichler A., Fatouros C., Lee H., Eisenhardt N. (2017). SUMO conjugation—A mechanistic view. Biomol. Concepts.

[23] Bonne-Andrea C., Kahli M., Mechali F., Lemaitre J.M., Bossis G., Coux O. (2013). SUMO2/3 modification of cyclin E contributes to the control of replication origin firing. Nat. Commun..

[24] Cubeñas-Potts C., Srikumar T., Lee C., Osula O., Subramonian D., Zhang X.D., Cotter R.J., Raught B., Matunis M.J. (2015). Identification of SUMO-2/3-modified proteins associated with mitotic chromosomes. Proteomics.

[25] Hong Y., Rogers R., Matunis M.J., Mayhew C.N., Goodson M.L., Park-Sarge O.K., Sarge K.D. (2001). Regulation of heat shock transcription factor 1 by stress-induced SUMO-1 modification. J. Biol. Chem..

[26] Ritho J., Arold S.T., Yeh E.T. (2015). A Critical SUMO1 Modification of LKB1 Regulates AMPK Activity during Energy Stress. Cell Rep..

[27] Zhang X.D., Goeres J., Zhang H., Yen T.J., Porter A.C., Matunis M.J. (2008). SUMO-2/3 modification and binding regulate the association of CENP-E with kinetochores and progression through mitosis. Mol. Cell.

[28] Gareau J.R., Lima C.D. (2010). The SUMO pathway: Emerging mechanisms that shape specificity, conjugation and recognition. Nat. Rev. Mol. Cell Biol..

[29] Sarge K.D., Park-Sarge O.K. (2009). Sumoylation and human disease pathogenesis. Trends Biochem. Sci..

[30] Rodriguez M.S., Dargemont C., Hay R.T. (2001). SUMO-1 conjugation in vivo requires both a consensus modification motif and nuclear targeting. J. Biol. Chem..

[31] Hay R.T. (2005). SUMO: A history of modification. Mol. Cell.

[32] Yunus A.A., Lima C.D. (2009). Structure of the Siz/PIAS SUMO E3 ligase Siz1 and determinants required for SUMO modification of PCNA. Mol. Cell.

[33] Werner A., Flotho A., Melchior F. (2012). The RanBP2/RanGAP1*SUMO1/Ubc9 complex is a multisubunit SUMO E3 ligase. Mol. Cell.

[34] Kagey M.H., Melhuish T.A., Wotton D. (2003). The polycomb protein Pc2 is a SUMO E3. Cell.

[35] Yang Y., Fiskus W., Yong B., Atadja P., Takahashi Y., Pandita T.K., Wang H.G., Bhalla K.N. (2013). Acetylated hsp70 and KAP1-mediated Vps34 SUMOylation is required for autophagosome creation in autophagy. Proc. Natl. Acad. Sci. USA.

[36] Rabellino A., Andreani C., Scaglioni P.P. (2017). The Role of PIAS SUMO E3-Ligases in Cancer. Cancer Res..

[37] Yeh E.T. (2009). SUMOylation and De-SUMOylation: Wrestling with life’s processes. J. Biol. Chem..

[38] Nayak A., Muller S. (2014). SUMO-specific proteases/isopeptidases: SENPs and beyond. Genome Biol..

[39] Shin E.J., Shin H.M., Nam E., Kim W.S., Kim J.H., Oh B.H., Yun Y. (2012). DeSUMOylating isopeptidase: A second class of SUMO protease. EMBO Rep..

[40] Schulz S., Chachami G., Kozaczkiewicz L., Winter U., Stankovic-Valentin N., Haas P., Hofmann K., Urlaub H., Ovaa H., Wittbrodt J. (2012). Ubiquitin-specific protease-like 1 (USPL1) is a SUMO isopeptidase with essential, non-catalytic functions. EMBO Rep..

[41] Hickey C.M., Wilson N.R., Hochstrasser M. (2012). Function and regulation of SUMO proteases. Nat. Rev. Mol. Cell Biol..

[42] Seeler J.S., Dejean A. (2003). Nuclear and unclear functions of SUMO. Nat. Rev. Mol. Cell Biol..

[43] Flotho A., Melchior F. (2013). Sumoylation: A regulatory protein modification in health and disease. Annu. Rev. Biochem..

[44] Yan D., Davis F.J., Sharrocks A.D., Im H.J. (2010). Emerging roles of SUMO modification in arthritis. Gene.

[45] Chang H.M., Yeh E.T.H. (2020). SUMO: From Bench to Bedside. Physiol. Rev..

[46] Berendsen A.D., Olsen B.R. (2015). Bone development. Bone.

[47] Salhotra A., Shah H.N., Levi B., Longaker M.T. (2020). Mechanisms of bone development and repair. Nat. Rev. Mol. Cell Biol..

[48] Li J., Lu D., Dou H., Liu H., Weaver K., Wang W., Li J., Yeh E.T.H., Williams B.O., Zheng L. (2018). Desumoylase SENP6 maintains osteochondroprogenitor homeostasis by suppressing the p53 pathway. Nat. Commun..

[49] Wan M., Cao X. (2005). BMP signaling in skeletal development. Biochem. Biophys. Res. Commun..

[50] Song B., Estrada K.D., Lyons K.M. (2009). Smad signaling in skeletal development and regeneration. Cytokine Growth Factor Rev..

[51] Shen J., Li S., Chen D. (2014). TGF-beta signaling and the development of osteoarthritis. Bone Res..

[52] Rahman M.S., Akhtar N., Jamil H.M., Banik R.S., Asaduzzaman S.M. (2015). TGF-beta/BMP signaling and other molecular events: Regulation of osteoblastogenesis and bone formation. Bone Res..

[53] Shimada K., Suzuki N., Ono Y., Tanaka K., Maeno M., Ito K. (2008). Ubc9 promotes the stability of Smad4 and the nuclear accumulation of Smad1 in osteoblast-like Saos-2 cells. Bone.

[54] Yukita A., Hosoya A., Ito Y., Katagiri T., Asashima M., Nakamura H. (2012). Ubc9 negatively regulates BMP-mediated osteoblastic differentiation in cultured cells. Bone.

[55] Kawano H., Sato T., Yamada T., Matsumoto T., Sekine K., Watanabe T., Nakamura T., Fukuda T., Yoshimura K., Yoshizawa T. (2003). Suppressive function of androgen receptor in bone resorption. Proc. Natl. Acad. Sci. USA.

[56] Wu J., Moverare-Skrtic S., Zhang F.P., Koskela A., Tuukkanen J., Palvimo J.J., Sipila P., Poutanen M., Ohlsson C. (2019). Androgen receptor SUMOylation regulates bone mass in male mice. Mol. Cell Endocrinol..

[57] Komori T. (2018). Runx2, an inducer of osteoblast and chondrocyte differentiation. Histochem. Cell Biol..

[58] Tang C.Y., Chen W., Luo Y., Wu J., Zhang Y., McVicar A., McConnell M., Liu Y., Zhou H.D., Li Y.P. (2020). Runx1 up-regulates chondrocyte to osteoblast lineage commitment and promotes bone formation by enhancing both chondrogenesis and osteogenesis. Biochem. J..

[59] Tang J., Xie J., Chen W., Tang C., Wu J., Wang Y., Zhou X.D., Zhou H.D., Li Y.P. (2020). Runt-related transcription factor 1 is required for murine osteoblast differentiation and bone formation. J. Biol. Chem..

[60] Wang Y., Feng Q., Ji C., Liu X., Li L., Luo J. (2017). RUNX3 plays an important role in mediating the BMP9-induced osteogenic differentiation of mesenchymal stem cells. Int. J. Mol. Med..

[61] Bauer O., Sharir A., Kimura A., Hantisteanu S., Takeda S., Groner Y. (2015). Loss of osteoblast Runx3 produces severe congenital osteopenia. Mol. Cell Biol..

[62] Nakashima K., Zhou X., Kunkel G., Zhang Z., Deng J.M., Behringer R.R., de Crombrugghe B. (2002). The novel zinc finger-containing transcription factor osterix is required for osteoblast differentiation and bone formation. Cell.

[63] Kim J.H., Jang J.W., Lee Y.S., Lee J.W., Chi X.Z., Li Y.H., Kim M.K., Kim D.M., Choi B.S., Kim J. (2014). RUNX family members are covalently modified and regulated by PIAS1-mediated sumoylation. Oncogenesis.

[64] Cai Z., Ding Y., Zhang M., Lu Q., Wu S., Zhu H., Song P., Zou M.H. (2016). Ablation of Adenosine Monophosphate-Activated Protein Kinase alpha1 in Vascular Smooth Muscle Cells Promotes Diet-Induced Atherosclerotic Calcification In Vivo. Circ. Res..

[65] Hosoya A., Yukita A., Ninomiya T., Hiraga T., Yoshiba K., Yoshiba N., Kasahara E., Nakamura H. (2013). Localization of SUMOylation factors and Osterix in odontoblast lineage cells during dentin formation and regeneration. Histochem. Cell Biol..

[66] Ali M.M., Yoshizawa T., Ishibashi O., Matsuda A., Ikegame M., Shimomura J., Mera H., Nakashima K., Kawashima H. (2007). PIASxbeta is a key regulator of osterix transcriptional activity and matrix mineralization in osteoblasts. J. Cell Sci..

[67] Liu H., Li J., Lu D., Li J., Liu M., He Y., Williams B.O., Li J., Yang T. (2018). Ginkgolic acid, a sumoylation inhibitor, promotes adipocyte commitment but suppresses adipocyte terminal differentiation of mouse bone marrow stromal cells. Sci. Rep..

[68] Zhang Y., Shao J., Wang Z., Yang T., Liu S., Liu Y., Fan X., Ye W. (2015). Growth differentiation factor 11 is a protective factor for osteoblastogenesis by targeting PPARgamma. Gene.

[69] Nayak A., Viale-Bouroncle S., Morsczeck C., Muller S. (2014). The SUMO-specific isopeptidase SENP3 regulates MLL1/MLL2 methyltransferase complexes and controls osteogenic differentiation. Mol. Cell.

[70] Ali A., Tyagi S. (2017). Diverse roles of WDR5-RbBP5-ASH2L-DPY30 (WRAD) complex in the functions of the SET1 histone methyltransferase family. J. Biosci..

[71] Bochynska A., Luscher-Firzlaff J., Luscher B. (2018). Modes of Interaction of KMT2 Histone H3 Lysine 4 Methyltransferase/COMPASS Complexes with Chromatin. Cells.

[72] Ernst P., Vakoc C.R. (2012). WRAD: Enabler of the SET1-family of H3K4 methyltransferases. Brief. Funct. Genomics.

[73] Liu C.F., Samsa W.E., Zhou G., Lefebvre V. (2017). Transcriptional control of chondrocyte specification and differentiation. Semin. Cell Dev. Biol..

[74] Decker R.S., Koyama E., Pacifici M. (2015). Articular Cartilage: Structural and Developmental Intricacies and Questions. Curr. Osteoporos. Rep..

[75] Akkiraju H., Nohe A. (2015). Role of Chondrocytes in Cartilage Formation, Progression of Osteoarthritis and Cartilage Regeneration. J. Dev. Biol..

[76] Sophia Fox A.J., Bedi A., Rodeo S.A. (2009). The basic science of articular cartilage: Structure, composition, and function. Sports Health.

[77] Akiyama H., Chaboissier M.C., Martin J.F., Schedl A., de Crombrugghe B. (2002). The transcription factor Sox9 has essential roles in successive steps of the chondrocyte differentiation pathway and is required for expression of Sox5 and Sox6. Genes. Dev..

[78] Lefebvre V., Behringer R.R., de Crombrugghe B. (2001). L-Sox5, Sox6 and Sox9 control essential steps of the chondrocyte differentiation pathway. Osteoarthr. Cartil..

[79] Liu C.F., Lefebvre V. (2015). The transcription factors SOX9 and SOX5/SOX6 cooperate genome-wide through super-enhancers to drive chondrogenesis. Nucleic. Acids Res..

[80] Hattori T., Eberspaecher H., Lu J., Zhang R., Nishida T., Kahyo T., Yasuda H., de Crombrugghe B. (2006). Interactions between PIAS proteins and SOX9 result in an increase in the cellular concentrations of SOX9. J. Biol. Chem..

[81] Liu J.A., Wu M.H., Yan C.H., Chau B.K., So H., Ng A., Chan A., Cheah K.S., Briscoe J., Cheung M. (2013). Phosphorylation of Sox9 is required for neural crest delamination and is regulated downstream of BMP and canonical Wnt signaling. Proc. Natl. Acad. Sci. USA.

[82] Pei H., Chen L., Liao Q.M., Wang K.J., Chen S.G., Liu Z.J., Zhang Z.C. (2018). SUMO-specific protease 2 (SENP2) functions as a tumor suppressor in osteosarcoma via SOX9 degradation. Exp. Ther. Med..

[83] Oh H.J., Kido T., Lau Y.F. (2007). PIAS1 interacts with and represses SOX9 transactivation activity. Mol. Reprod. Dev..

[84] Zuo C., Wang L., Kamalesh R.M., Bowen M.E., Moore D.C., Dooner M.S., Reginato A.M., Wu Q., Schorl C., Song Y. (2018). SHP2 regulates skeletal cell fate by modifying SOX9 expression and transcriptional activity. Bone Res..

[85] Grossmann K.S., Rosario M., Birchmeier C., Birchmeier W. (2010). The tyrosine phosphatase Shp2 in development and cancer. Adv. Cancer Res..

[86] Neel B.G., Gu H., Pao L. (2003). The ’Shp’ing news: SH2 domain-containing tyrosine phosphatases in cell signaling. Trends Biochem. Sci..

[87] Fernandez-Lloris R., Osses N., Jaffray E., Shen L.N., Vaughan O.A., Girwood D., Bartrons R., Rosa J.L., Hay R.T., Ventura F. (2006). Repression of SOX6 transcriptional activity by SUMO modification. FEBS Lett..

[88] Choi H.J., Kwon S., Kim D.W. (2016). A post-translational modification cascade employing HDAC9-PIASy-RNF4 axis regulates chondrocyte hypertrophy by modulating Nkx3.2 protein stability. Cell Signal.

[89] Deng L., Ren R., Liu Z., Song M., Li J., Wu Z., Ren X., Fu L., Li W., Zhang W. (2019). Stabilizing heterochromatin by DGCR8 alleviates senescence and osteoarthritis. Nat. Commun..

[90] Zhu C., Chen C., Huang J., Zhang H., Zhao X., Deng R., Dou J., Jin H., Chen R., Xu M. (2015). SUMOylation at K707 of DGCR8 controls direct function of primary microRNA. Nucleic. Acids Res..

[91] Kobayashi T., Papaioannou G., Mirzamohammadi F., Kozhemyakina E., Zhang M., Blelloch R., Chong M.W. (2015). Early postnatal ablation of the microRNA-processing enzyme, Drosha, causes chondrocyte death and impairs the structural integrity of the articular cartilage. Osteoarthr. Cartil..

[92] Li S., Wang M., Ao X., Chang A.K., Yang C., Zhao F., Bi H., Liu Y., Xiao L., Wu H. (2013). CLOCK is a substrate of SUMO and sumoylation of CLOCK upregulates the transcriptional activity of estrogen receptor-α. Oncogene.

[93] Liang C., Liu Z., Song M., Li W., Wu Z., Wang Z., Wang Q., Wang S., Yan K., Sun L. (2021). Stabilization of heterochromatin by CLOCK promotes stem cell rejuvenation and cartilage regeneration. Cell Res..

[94] Chen D., Shen J., Zhao W., Wang T., Han L., Hamilton J.L., Im H.J. (2017). Osteoarthritis: Toward a comprehensive understanding of pathological mechanism. Bone Res..

[95] arcOGEN Consortium, arcOGEN Collaborators (2012). Identification of new susceptibility loci for osteoarthritis (arcOGEN): A genome-wide association study. Lancet.

[96] Johnson K., Reynard L.N., Loughlin J. (2015). Functional characterisation of the osteoarthritis susceptibility locus at chromosome 6q14.1 marked by the polymorphism rs9350591. BMC Med. Genet..

[97] Miranda K.J., Loeser R.F., Yammani R.R. (2010). Sumoylation and nuclear translocation of S100A4 regulate IL-1beta-mediated production of matrix metalloproteinase-13. J. Biol. Chem..

[98] Daouti S., Latario B., Nagulapalli S., Buxton F., Uziel-Fusi S., Chirn G.W., Bodian D., Song C., Labow M., Lotz M. (2005). Development of comprehensive functional genomic screens to identify novel mediators of osteoarthritis. Osteoarthr. Cartil..

[99] Sun T., Gao F., Lin X., Yu R., Zhao Y., Luan J., Li H., Song M. (2014). alpha-Lipoic acid (alpha-LA) inhibits the transcriptional activity of interferon regulatory factor 1 (IRF-1) via SUMOylation. Toxicol. Vitr..

[100] Im H.J., Sharrocks A.D., Lin X., Yan D., Kim J., van Wijnen A.J., Hipskind R.A. (2009). Basic fibroblast growth factor induces matrix metalloproteinase-13 via ERK MAP kinase-altered phosphorylation and sumoylation of Elk-1 in human adult articular chondrocytes. Open. Access Rheumatol..

[101] Ash P., Loutit J.F., Townsend K.M. (1980). Osteoclasts derived from haematopoietic stem cells. Nature.

[102] Feng X., Teitelbaum S.L. (2013). Osteoclasts: New Insights. Bone Res..

[103] Zhang Y., Yang K., Yang J., Lao Y., Deng L., Deng G., Yi J., Sun X., Wang Q. (2020). SENP3 Suppresses Osteoclastogenesis by De-conjugating SUMO2/3 from IRF8 in Bone Marrow-Derived Monocytes. Cell Rep..

[104] Hikata T., Takaishi H., Takito J., Hakozaki A., Furukawa M., Uchikawa S., Kimura T., Okada Y., Matsumoto M., Yoshimura A. (2009). PIAS3 negatively regulates RANKL-mediated osteoclastogenesis directly in osteoclast precursors and indirectly via osteoblasts. Blood.

[105] Palvimo J.J. (2007). PIAS proteins as regulators of small ubiquitin-related modifier (SUMO) modifications and transcription. Biochem. Soc. Trans..

[106] Elliott A.M., Evans J.A., Chudley A.E. (2005). Split hand foot malformation (SHFM). Clin. Genet..

[107] Ianakiev P., Kilpatrick M.W., Toudjarska I., Basel D., Beighton P., Tsipouras P. (2000). Split-hand/split-foot malformation is caused by mutations in the p63 gene on 3q27. Am. J. Hum. Genet..

[108] Van Bokhoven H., Hamel B.C., Bamshad M., Sangiorgi E., Gurrieri F., Duijf P.H., Vanmolkot K.R., van Beusekom E., van Beersum S.E., Celli J. (2001). p63 Gene mutations in eec syndrome, limb-mammary syndrome, and isolated split hand-split foot malformation suggest a genotype-phenotype correlation. Am. J. Hum. Genet..

[109] Ghioni P., D’Alessandra Y., Mansueto G., Jaffray E., Hay R.T., La Mantia G., Guerrini L. (2005). The protein stability and transcriptional activity of p63alpha are regulated by SUMO-1 conjugation. Cell Cycle.

[110] Huang Y.P., Wu G., Guo Z., Osada M., Fomenkov T., Park H.L., Trink B., Sidransky D., Fomenkov A., Ratovitski E.A. (2004). Altered sumoylation of p63alpha contributes to the split-hand/foot malformation phenotype. Cell Cycle.

[111] Ranieri M., Vivo M., De Simone M., Guerrini L., Pollice A., La Mantia G., Calabro V. (2018). Sumoylation and ubiquitylation crosstalk in the control of DeltaNp63alpha protein stability. Gene.

[112] Mossey P.A., Little J., Munger R.G., Dixon M.J., Shaw W.C. (2009). Cleft lip and palate. Lancet.

[113] Alkuraya F.S., Saadi I., Lund J.J., Turbe-Doan A., Morton C.C., Maas R.L. (2006). SUMO1 haploinsufficiency leads to cleft lip and palate. Science.

[114] Song T., Li G., Jing G., Jiao X., Shi J., Zhang B., Wang L., Ye X., Cao F. (2008). SUMO1 polymorphisms are associated with non-syndromic cleft lip with or without cleft palate. Biochem. Biophys. Res. Commun..

[115] Mostowska A., Hozyasz K.K., Wojcicki P., Biedziak B., Paradowska P., Jagodzinski P.P. (2010). Association between genetic variants of reported candidate genes or regions and risk of cleft lip with or without cleft palate in the polish population. Birth. Defects Res. A Clin. Mol. Teratol..

[116] Carter T.C., Molloy A.M., Pangilinan F., Troendle J.F., Kirke P.N., Conley M.R., Orr D.J., Earley M., McKiernan E., Lynn E.C. (2010). Testing reported associations of genetic risk factors for oral clefts in a large Irish study population. Birth Defects Res. A Clin. Mol. Teratol..

[117] Jia Z.L., Li Y., Meng T., Shi B. (2010). Association between polymorphisms at small ubiquitin-like modifier 1 and nonsyndromic orofacial clefts in Western China. DNA Cell Biol..

[118] Pauws E., Stanier P. (2007). FGF signalling and SUMO modification: New players in the aetiology of cleft lip and/or palate. Trends Genet..

[119] Dobreva G., Dambacher J., Grosschedl R. (2003). SUMO modification of a novel MAR-binding protein, SATB2, modulates immunoglobulin mu gene expression. Genes. Dev..

[120] Antonio Urrutia G., Ramachandran H., Cauchy P., Boo K., Ramamoorthy S., Boller S., Dogan E., Clapes T., Trompouki E., Torres-Padilla M.E. (2021). ZFP451-mediated SUMOylation of SATB2 drives embryonic stem cell differentiation. Genes. Dev..

[121] Gupta V., Bei M. (2006). Modification of Msx1 by SUMO-1. Biochem. Biophys. Res. Commun..

[122] Song Y.J., Lee H. (2011). PIAS1 negatively regulates ubiquitination of Msx1 homeoprotein independent of its SUMO ligase activity. Mol. Cells.

[123] Andreou A.M., Pauws E., Jones M.C., Singh M.K., Bussen M., Doudney K., Moore G.E., Kispert A., Brosens J.J., Stanier P. (2007). TBX22 missense mutations found in patients with X-linked cleft palate affect DNA binding, sumoylation, and transcriptional repression. Am. J. Hum. Genet..

[124] Kaiser F.J., Ludecke H.J., Weger S. (2007). SUMOylation modulates transcriptional repression by TRPS1. Biol. Chem..

[125] Guo Q., Wang Y., Xu D., Nossent J., Pavlos N.J., Xu J. (2018). Rheumatoid arthritis: Pathological mechanisms and modern pharmacologic therapies. Bone Res..

[126] (2018). Rheumatoid arthritis. Nat. Rev. Dis. Primers.

[127] Franz J.K., Pap T., Hummel K.M., Nawrath M., Aicher W.K., Shigeyama Y., Muller-Ladner U., Gay R.E., Gay S. (2000). Expression of sentrin, a novel antiapoptotic molecule, at sites of synovial invasion in rheumatoid arthritis. Arthritis Rheum..

[128] Lao M., Shi M., Zou Y., Huang M., Ye Y., Qiu Q., Xiao Y., Zeng S., Liang L., Yang X. (2016). Protein Inhibitor of Activated STAT3 Regulates Migration, Invasion, and Activation of Fibroblast-like Synoviocytes in Rheumatoid Arthritis. J. Immunol..

[129] Lao M., Zhan Z., Li N., Xu S., Shi M., Zou Y., Huang M., Zeng S., Liang L., Xu H. (2019). Role of small ubiquitin-like modifier proteins-1 (SUMO-1) in regulating migration and invasion of fibroblast-like synoviocytes from patients with rheumatoid arthritis. Exp. Cell Res..

[130] Wang C., Xiao Y., Lao M., Wang J., Xu S., Li R., Xu X., Kuang Y., Shi M., Zou Y. (2020). Increased SUMO-activating enzyme SAE1/UBA2 promotes glycolysis and pathogenic behavior of rheumatoid fibroblast-like synoviocytes. JCI Insight.

[131] Meinecke I., Cinski A., Baier A., Peters M.A., Dankbar B., Wille A., Drynda A., Mendoza H., Gay R.E., Hay R.T. (2007). Modification of nuclear PML protein by SUMO-1 regulates Fas-induced apoptosis in rheumatoid arthritis synovial fibroblasts. Proc. Natl. Acad. Sci. USA.

[132] Maciejewska-Rodrigues H., Karouzakis E., Strietholt S., Hemmatazad H., Neidhart M., Ospelt C., Gay R.E., Michel B.A., Pap T., Gay S. (2010). Epigenetics and rheumatoid arthritis: The role of SENP1 in the regulation of MMP-1 expression. J. Autoimmun..

[133] Li F., Li X., Kou L., Li Y., Meng F., Ma F. (2014). SUMO-conjugating enzyme UBC9 promotes proliferation and migration of fibroblast-like synoviocytes in rheumatoid arthritis. Inflammation.

[134] Frank S., Peters M.A., Wehmeyer C., Strietholt S., Koers-Wunrau C., Bertrand J., Heitzmann M., Hillmann A., Sherwood J., Seyfert C. (2013). Regulation of matrixmetalloproteinase-3 and matrixmetalloproteinase-13 by SUMO-2/3 through the transcription factor NF-kappaB. Ann. Rheum. Dis..

[135] Vatsyayan J., Qing G., Xiao G., Hu J. (2008). SUMO1 modification of NF-kappaB2/p100 is essential for stimuli-induced p100 phosphorylation and processing. EMBO Rep..

[136] Liu J., Sha M., Wang Q., Ma Y., Geng X., Gao Y., Feng L., Shen Y., Shen Y. (2015). Small ubiquitin-related modifier 2/3 interacts with p65 and stabilizes it in the cytoplasm in HBV-associated hepatocellular carcinoma. BMC Cancer.

[137] Leidner J., Voogdt C., Niedenthal R., Moller P., Marienfeld U., Marienfeld R.B. (2014). SUMOylation attenuates the transcriptional activity of the NF-kappaB subunit RelB. J. Cell Biochem..

[138] Liu J., Wu Z., Han D., Wei C., Liang Y., Jiang T., Chen L., Sha M., Cao Y., Huang F. (2020). Mesencephalic Astrocyte-Derived Neurotrophic Factor Inhibits Liver Cancer Through Small Ubiquitin-Related Modifier (SUMO)ylation-Related Suppression of NF-kappaB/Snail Signaling Pathway and Epithelial-Mesenchymal Transition. Hepatology.

[139] Whelan J.S., Davis L.E. (2018). Osteosarcoma, Chondrosarcoma, and Chordoma. J. Clin. Oncol..

[140] Ritter J., Bielack S.S. (2010). Osteosarcoma. Ann. Oncol..

[141] Seeler J.S., Dejean A. (2017). SUMO and the robustness of cancer. Nat. Rev. Cancer.

[142] Eifler K., Vertegaal A.C.O. (2015). SUMOylation-Mediated Regulation of Cell Cycle Progression and Cancer. Trends Biochem. Sci..

[143] Zhang D., Yu K., Yang Z., Li Y., Ma X., Bian X., Liu F., Li L., Liu X., Wu W. (2018). Silencing Ubc9 expression suppresses osteosarcoma tumorigenesis and enhances chemosensitivity to HSV-TK/GCV by regulating connexin 43 SUMOylation. Int. J.Oncol..

[144] Liu F., Li L., Li Y., Ma X., Bian X., Liu X., Wang G., Zhang D. (2018). Overexpression of SENP1 reduces the stemness capacity of osteosarcoma stem cells and increases their sensitivity to HSVtk/GCV. Int. J. Oncol..

[145] Huang Z., Barker D., Gibbins J.M., Dash P.R. (2018). Talin is a substrate for SUMOylation in migrating cancer cells. Exp. Cell Res..

[146] Wang K., Zhang X.C. (2014). Inhibition of SENP5 suppresses cell growth and promotes apoptosis in osteosarcoma cells. Exp. Ther. Med..

[147] Wang J., Ni J., Yi S., Song D., Ding M. (2016). Protein inhibitor of activated STAT xalpha depresses cyclin D and cyclin D kinase, and contributes to the inhibition of osteosarcoma cell progression. Mol. Med. Rep..

[148] Luo P., Yang X., Ying M., Chaudhry P., Wang A., Shimada H., May W.A., Adams G.B., Mock D., Triche T.J. (2010). Retinoid-suppressed phosphorylation of RARalpha mediates the differentiation pathway of osteosarcoma cells. Oncogene.

[149] Zhou Q., Zhang L., Chen Z., Zhao P., Ma Y., Yang B., He Q., Ying M. (2014). Small ubiquitin-related modifier-1 modification regulates all-trans-retinoic acid-induced differentiation via stabilization of retinoic acid receptor alpha. FEBS J..

[150] Wang X., Liang X., Liang H., Wang B. (2018). SENP1/HIF-1alpha feedback loop modulates hypoxia-induced cell proliferation, invasion, and EMT in human osteosarcoma cells. J. Cell Biochem..

[151] Wang L., Wu J., Song S., Chen H., Hu Y., Xu B., Liu J. (2021). Plasma Exosome-Derived Sentrin SUMO-Specific Protease 1: A Prognostic Biomarker in Patients With Osteosarcoma. Front. Oncol..

[152] Zhu L., Plow E.F., Qin J. (2021). Initiation of focal adhesion assembly by talin and kindlin: A dynamic view. Protein Sci..

[153] Kroonen J.S., Kruisselbrink A.B., Briaire-de Bruijn I.H., Olaofe O.O., Bovee J., Vertegaal A.C.O. (2021). SUMOylation Is Associated with Aggressive Behavior in Chondrosarcoma of Bone. Cancers.

[154] Brackett C.M., Blagg B.S.J. (2021). Current Status of SUMOylation Inhibitors. Curr. Med. Chem..

[155] Kukkula A., Ojala V.K., Mendez L.M., Sistonen L., Elenius K., Sundvall M. (2021). Therapeutic Potential of Targeting the SUMO Pathway in Cancer. Cancers.

